# Transactions of the Pathological Society of Philadelphia

**Published:** 1839-12-14

**Authors:** 


### TRANSACTIONS OF THE PATHOLOGICAL SOCIETY OF PHILADELPHIA.

*December 1st*, 1839.

The President, Dr. Gerhard, in the Chair.

Dr. Stewardson presented a specimen of *Diseased Heart* and *Liver*, and read the following account of the case:

### *Case of Hypertrophy and Dilatation of the Heart, with Disease of the Liver.*

The subject of the following case was a private patient of Dr. Caspar Morris, to whom I am indebted, with slight exceptions, for the following account of the symptoms, &c., which I give nearly in his own words.

John C——, aged 76 years, has been in the habit of consulting me for some five or six years on account of some dyspeptic symptoms,—his chief suffering being from acidity of stomach, for the relief of which I have been accustomed to recommend attention to his diet, and the use of soda, with occasional recourse to blue mass, rhubarb, and soda. He followed the business of a labourer in a stone yard, chiefly engaged in sawing marble. In the autumn of 1838 he had a smart attack of rheumatic fever, with pain, and swelling of the joints and muscles, especially of the arms. In the course of the attack there was considerable oppression in breathing, which was relieved, together with the general symptoms, by *free* depletion and purging, first with colchicum, and then with powdered guaiacum. He resumed his occupation in November, and continued it throughout the winter and summer, interrupted only by an attack of what I supposed at the time to be pleurisy, affecting the lower lobe of the right lung, which occurred about the month of May or June, accompanied by pain in right side, along margin of false ribs, slight cough, full pulse, hot skin, &c., which was treated by very abundant depletion, both general and local, and blistering over the seat of pain. From this attack he never entirely recovered, though he was able to resume his labour, and continue it until the month of August. In this month I was sent for suddenly early on a Monday morning, and found him suffering from intense pain under the right clavicle, about the middle of the bone, with laboured respiration, and a full pulse. He stated, that the preceding day, as he came home from church, he had suddenly been seized with this pain and difficulty of breathing, to such a degree, that it was only by great exertion he had been able to walk home, stopping repeatedly on the way. The sounds of respiration, I remember, were perfect, and there was no marked flatness on percussion on any part of the thorax. The action of the heart was laboured, but I cannot recall any recollection of the nature of the sound, or the force of the impulse. Bleeding, cupping, and blistering, were resorted to without any thing beyond temporary relief. There was diminution of the secretion of urine, and it was generally very high coloured. In September I examined his chest attentively: the respiratory sounds were very distinct in the left lung, and the resonance greater than in the right, which appeared, however, in a normal condition. There was, then, so far as my memory serves me, no unnatural sound in the action of the heart, but a marked want of strength in the impulse. There was an inability to lie with the head low; hard and dry cough. The treatment consisted in frequent bleeding, which always relieved him for the time, and diuretics. The skin was pale, and the countenance always anxious, though his mind was cheerful. The relief afforded by bleeding gradually diminished, and the strength failed; under these circumstances I suspended treatment, recommending mild diet and quiet, as the only means of prolonging his life and affording him ease. He was, in fact, compelled to quiet, from the great difficulty of breathing produced by the attempt at exertion. In the month of October Dr. Gerhard saw him with me, and examined his symptoms, which, in his opinion, designated disease of the liver, with some slight affection of the heart. The difficulty of breathing he was disposed to attribute to emphysema. Early in November the symptoms were all aggravated, accompanied by effusion into the abdomen, and swelling of the legs and thighs, with coldness of the hands and lividity of the surface. Dr. Pennock now saw him, and made an examination of the chest. The left side was rather larger than the right; there was dulness on percussion over the lower half of the right side; at left, the præcordial dulness was more extended than natural, except towards the upper part of the chest, where the percussion was very resonant for about three inches under clavicle; there was no perceptible impulse of the heart; both sounds could be heard distinctly and naturally over the region of the pulmonary artery, whilst along the line of the sternum, at its upper part, as well as at the epigastrium, there was a distinct and prolonged rasping accompanying the first sound; elsewhere over the præcordial region, this roughened sound was heard faintly, if at all; there was great action of the veins of the neck, especially on the right side. The patient was not naturally subject to short breath; and before his recent attack, could run as well as any one. No hæmoptysis; no expectoration since attack in August.

On the morning of November 19th, between twenty-four and forty-eight hours after death, I made the examination in company with Dr. Morris.

*Abdomen.*—Moderate effusion, say from one to two pints of yellow serum in cavity; general aspect of peritoneal covering healthy.

*Liver* smaller than natural; left lobe does not extend more than two or three inches to left of suspensory ligament; coats thickened and opaque, and divisible in layers for several inches in extent over upper surface of great lobe, and perhaps elsewhere, as the whole liver was not removed from the body. Externally, also, it is slightly rough to the touch in places, and has a mottled aspect. Proper coat easily separated from the substance; being cut into, the surface every where presents a spotted appearance,—these spots being from half a line to a line in diameter, of a more or less bright yellow, circular, with a central point, and surrounded either in part or entirely by a reddish brown substance. These yellow spots evidently answer to the substance of the acini.

The *gall bladder* contains a considerable quantity of thick, tenacious, very dark coloured bile, having a deep orange tint when spread out in a thin layer. No obstruction found in hepatic duct, or ductus communis.

*Stomach* of moderate size, containing about six ounces of a thin, dirty white fluid. Mucous membrane generally pale, thinned and softened in the great cul de sac.

The mucous membrane of the small and large intestines was not examined throughout; but in places where cut into nothing remarkable was observed, except that in large intestine it was covered with a good deal of glairy and tenacious mucus.

*Spleen* and *kidneys* natural.

*Pancreas* also, except that it seems to be more dense than natural.

*Chest.*—Upon opening the chest, the upper lobe of the left lung is found to extend to the median line, and almost overlap that of the opposite side, and extending downwards along same line for about three inches below the end of the clavicle; it hardly collapses at all, being applied very nearly to the walls of the chest anteriorly. Left lung collapses moderately; about a quart of bloody serum in each pleural cavity, mixed on the right side, at least, with some flocculi of lymph; no adhesion any where.

*Right lung.*—Air vesicles of upper lobe dilated principally at the anterior part, where the border is much thickened; tissue otherwise healthy, supple, and crepitating; lower lobe much congested, of a uniform deep colour.

Right lung, upper lobe, healthy, except some slight dilatations of the cells. Upon cutting into the lower lobe, a number of spots, of a deep black colour, like coagulated blood, were observed; they varied in size, being from half an inch to an inch and a half in diameter, more solid and firm than the surrounding tissue, distinctly circumscribed, presenting no traces of organization, but easily broken down by the scalpel, when some fibres of the natural tissue of the lung could be seen stretching across them; tissue around presented nothing remarkable; no tubercles observed any where.

*Pericardium* contains a small spoonful of serum; internal surface smooth; no adhesions.

*Heart* much enlarged, fully twice the size of the fist of the subject; walls firm; length from apex to base along septum four inches; breadth across base five inches; large black coagula in both ventricles; external membrane of heart smooth, presenting several whitish, and more or less opaque patches, accompanied with thickening; unusual quantity of fatty matter over right ventricle, the cavity of which is nearly twice its natural size; walls one and three-quarter lines near base, and one and a quarter near the point; internal membrane smooth, without opacity; auriculo-ventricular orifice four inches eight lines; mitral valve natural; *right auricle* much enlarged; columnæ carneæ proportionally developed; lining membrane presenting nothing remarkable.

*Pulmonary Artery.*—Circumference at orifice three inches eight lines; valves supple, translucent and free, of a rose tint; lining membrane of artery smooth, and also of a rose tint with here and there a few minute whitish specks.

*Left Ventricle.*—Walls six lines near base, and four near the point; capacity doubled; internal membrane slightly opaque over a space two inches in diameter immediately bordering upon the aortic valves, which are also thickened and opaque, but preserve their suppleness in a great measure.

*Aorta.*—Circumference at orifice three and a half inches; immediately above valves and nearly on a line with their upper margin, is a ridge of bony matter extending nearly two-thirds round the circumference of the artery; this bony ridge is only a few lines broad, and in one part is elevated about two lines above the surrounding surface, its margin being very irregular and broken; elsewhere it is less elevated, and its surface more or less smooth; in its neighbourhood are a few small patches of bony matter, as well as about the origin of the vessels at the arch. Internal surface of aorta elsewhere smooth; circumference an inch above orifice, three and three-quarter inches. Left auricle enlarged; internal membrane opaque, white; circumference of auriculo-ventricular orifice, three inches seven lines; mitral valve thickened, but still pretty supple, except along adherent border near aortic orifice, where there are several deposits of matter apparently cartilaginous.

*Remarks.*—It is impossible in the above case, to trace with certainty the origin of the disease of the heart, to the attack of acute rheumatism which occurred about a year before the patient's death, since, previous to that period, a sufficiently accurate examination was not made to determine positively whether the organ was or was not the seat of organic disease. If any general symptoms of such disease existed, however, they were slight, and during the attack of rheumatism, the patient suffered under great difficulty of breathing, together, probably with other symptoms not contained in the notes, and referrible to the chest,—as Dr. Morris supposed the patient to be labouring at the time under cardiac inflammation. The existence of this inflammation is farther confirmed by the evidences found after death, viz. the partial thickening of the lining membrane of the left ventricle, &c. If this view of the case be correct, it adds another to the many which have been adduced within a few years, illustrative of the connection, on the one hand between cardiac inflammation, and articular rheumatism as its cause, and on the other, between the same inflammation and certain organic changes of the heart, as hypertrophy, &c., as its effect.

The connection between the physical signs and the condition of the organ, is particularly worthy of attention. The prolonged rasping sound heard along the line of the sternum, especially at its upper part, was evidently occasioned by the irregular ridge of bony matter stretching nearly around the artery on a level with the upper margin of its valves. On the contrary, both sounds were heard perfectly natural, farther to the left over the region of the pulmonary valves, which were found perfectly healthy. The absence of impulse was probably owing to the great enlargement, without *absolute* thickening of the walls of the heart. In connection with the chronic alteration of the liver, it will be recollected that the patient laboured about five months before his death under an acute attack of disease, characterized by pain along the margin of the false ribs of the right side, slight cough and fever, and which I think we are justified in concluding, was probably hepatitis, as no adhesions of the pleura were found after death. The alteration of the liver in the present instance was essentially the same with that presented by me at the last meeting, and also with that found in the case of Margaret Eretel, who was under my charge at the Pennsylvania Hospital till within a few weeks of her death, and reported by Dr. Stillé. Each of these patients had laboured under symptoms of acute inflammation in the region of the liver; and in the case of Margaret Eretel, especially, I think that the chronic alteration of the organ was evidently referrible to the previous acute attack as its immediate cause, followed as it was so soon by ascites unaccompanied by general dropsy. The most that could be said of the influence of disease of the heart, even supposing that it existed in this instance, which is doubtful, previous to the attack of hepatitis, would be that it operated as a predisposing cause to the latter, to which we would still be obliged to recur as the direct and immediate cause of the chronic alteration of the liver. In other words, we must admit that this alteration was probably the result of inflammation. That organic disease of the heart is productive of congestion of the liver is certain, but the lesion before us is not mere congestion, but a chronic change of structure, characterized by hardness and paleness, or yellowness of the acini. However, I am not disposed to deny, even here, the influence of disease of the heart as a predisposing cause, a supposition indeed rendered highly probable by the frequent simultaneous occurrence of the two lesions. But admitting all this, and even granting that the lesion of the liver is in some cases the result of that of the heart without the intervention of any other cause, the opinion above expressed in reference to its nature, would not in the least degree be invalidated. We should still be justified in regarding it as a chronic inflammation, mostly traceable to an acute attack, though, sometimes the gradual result of long continued congestion. This subject is of practical importance, because, if we look upon the disease of the liver as mostly independent to a certain extent, and in some cases entirely of disease of the heart, we should feel ourselves called upon to make use of more active measures for its removal, than if we regarded it as a mere appendage of that of the heart.

---

Dr. Thomas F. Betton presented a specimen of *Aneurism of the Aorta*, in which the Aneurismal Tumor had caused the absorption of the ribs and presented externally.

